# The relationship between dissociation and panic symptoms in adolescence and the exploration of potential mediators

**DOI:** 10.1002/jcv2.12202

**Published:** 2023-10-11

**Authors:** Lottie Shipp, Alisa Musatova, Emma Černis, Polly Waite

**Affiliations:** ^1^ Department of Experimental Psychology University of Oxford Oxford UK; ^2^ School of Psychology University of Birmingham Birmingham UK; ^3^ Department of Psychiatry University of Oxford Oxford UK

**Keywords:** alexithymia, anxiety, appraisals, cognitions, dissociation, emotion regulation

## Abstract

**Background:**

Dissociative experiences have been linked to panic symptoms in adolescents, yet the nature of the association remains unclear.

**Methods:**

In the present study, we investigated the longitudinal relationship between dissociative experiences (focusing on the felt sense of anomaly subtype) and panic, as well as the potential mediating roles of emotion regulation strategies (expressive suppression and cognitive reappraisal), alexithymia, and cognitive appraisals of dissociation. Four thousand five hundred one adolescents aged 13–18 years were recruited via social media advertising to take part in an online survey at two timepoints, 1 month apart.

**Results:**

Analysis of 421 datasets found a significant positive relationship between initial dissociative experiences and panic symptoms reported 1 month later. This was mediated by the emotion regulation strategy of cognitive reappraisal, and cognitive appraisals of dissociation. These two variables were no longer significant mediators when controlling for panic symptoms at the first time point, likely due to the stability of panic symptoms across both assessments. Neither alexithymia nor expressive suppression were significant mediators.

**Conclusions:**

Thus, dissociative experiences that are persistently misinterpreted in a catastrophic manner may lead to escalating anxiety and panic symptoms, which could in turn heighten and maintain the feared dissociation sensation. These results indicate that dissociative experiences are associated with panic symptoms in adolescence, with cognitive appraisals of dissociation and cognitive reappraisal playing a role in this relationship.

**Key points**

- Dissociation has been linked to panic symptoms in adolescents, yet little is known about this relationship and the roles of any potential mediators.
- This study found a significant positive relationship between dissociative experiences and panic symptoms reported 1 month later.
- This longitudinal relationship was mediated by the emotion regulation strategy of cognitive reappraisal, and cognitive appraisals of dissociation.
- Mediating relationships were no longer significant when controlling for initial panic symptoms and this is likely due to their stability across both assessment points.
- Results align with existing models, which state that dissociative experiences, when misinterpreted in a catastrophic manner, may lead to escalating panic symptoms.
- Future studies should aim to further explore the temporal relationship between these variables, ideally in clinical populations of adolescents with panic disorder, who may experience qualitatively different panic and dissociation symptoms.

## INTRODUCTION

Adolescence is a time of life associated with an increased risk for psychopathology (Powers & Casey, 2015), with 1%–3% of 11–19 year olds meeting diagnostic criteria for panic disorder (Vizard et al., 2018). This risk appears to have been exacerbated by the COVID‐19 pandemic with increased rates of mental health problems amongst adolescents (NHS Digital, 2020) from pre‐pandemic levels, and therefore it is possible that the prevalence of panic disorder or panic attacks has also recently increased. Dissociative feelings (e.g., depersonalisation and derealisation–feelings of disconnection from oneself and the external world, respectively) are commonly reported by adolescents with high levels of panic symptoms or panic disorder (Achiam‐Montal et al., 2013; Doerfler et al., 2007). The multidimensional approach (e.g., Holmes et al., 2005) suggests that clusters of dissociative phenomenological experiences form separable constructs. One such subtype is ‘felt sense of anomaly’‐type dissociation (FSA‐dissociation; Černis et al., 2021): dissociative experiences that share a subjective sensation of oddness or strangeness. This can occur with respect to perception, the physical body, mental content, or the experience of oneself and the surrounding environment, and may manifest as feelings of detachment, unfamiliarity, or lack of control.

There is no existing model that explains dissociation with respect to panic in adolescents. However, there are cognitive models of panic (Clark, 1986) and depersonalisation disorder (DPD; Hunter et al., 2003), and a tentative model of FSA‐dissociation (Černis et al., 2022), in adults. These propose that catastrophic misinterpretations of benign physiological or psychological symptoms set in motion a cycle of escalating anxiety and heightened dissociation or panic symptoms. Given the relationship between dissociation and panic symptoms in adults (Cassano et al., 1989; Marshall et al., 2000; Seguí et al., 2000), and that dissociation is commonly experienced by young people with panic disorder (Doerfler et al., 2007), dissociative experiences may plausibly trigger panic symptoms in adolescents when misinterpreted as indicative of impending catastrophe (Hunter et al., 2003; Černis et al., 2020). This is supported by the finding that the relationship between adolescent trait anxiety and dissociation (including FSA‐dissociation) is mediated by negative cognitive appraisals of dissociation (Lofthouse et al., 2023). However, to our knowledge, there are currently no longitudinal studies of adolescents that examine the association between dissociation and panic symptoms.

One potential mediator of the relationship between panic symptoms and dissociation is how the individual makes sense of their dissociative experiences—their cognitive appraisals of dissociation. This construct relates to the content of the interpretation and the specific beliefs that the individual holds regarding the dissociative experience (Černis et al., 2020). Where dissociation is associated with psychopathology, cognitive appraisals of dissociation are typically negative—for example, feeling dissociated might be interpreted as a sign of losing control of one's body or mind.

It is also possible that any relationship between these constructs in adolescents may be mediated by emotion regulation: the ways in which one can exert control over the experience and expression of their emotions (Gross, 1998; Gross & John, 2003). Adults with panic disorder, fearing that their anxiety will be noticed by others, have been shown to suppress their expression of emotion (Strauss et al., 2019). Although beneficial in certain situations, habitual and inflexible expressive suppression is maladaptive (Gross, 2002), with the use of this strategy predictive of subsequent panic symptom severity (Strauss et al., 2019) and greater depersonalisation or derealisation (Tibubos et al., 2018). Furthermore, adults with panic disorder demonstrate stronger control of expression of emotion compared to community participants, and a greater tendency to ‘bottle up’ their feelings (Baker et al., 2004). This could be considered a safety behaviour from a cognitive‐behavioural perspective, as attempts to control emotions and bodily sensations with the aim of reducing anxiety have the paradoxical effect of maintaining or increasing symptoms (Aafjes‐van Doorn et al., 2019; Clark, 1999). Additionally, Černis et al. (2022a) suggest that individuals vulnerable to FSA‐dissociation may view emotions as internal threats and believe that they have little ability to cope with heightened affect. Thus, it is plausible that use of expressive suppression is motivated by affect intolerance–however, given the cross‐sectional nature of these studies, it is unclear whether habitual expressive suppression causes dissociation or if dissociation hinders emotion regulation. The two may even be reciprocal risk factors for each other, as both are associated with difficulties responding adaptively to internal sensations and/or emotional states (Cavicchioli et al., 2021).

A second, adaptive emotion regulation strategy is cognitive reappraisal: changing the way one construes a situation to alter its emotional impact (Gross & John, 2003)–for example, seeing panic‐related sensations as normal not harmful. Cognitive reappraisal of sensations is central to cognitive‐behavioural approaches to panic disorder (Clark, 1986) and DPD (Hunter et al., 2003), and in adults it is negatively associated with both anxiety (Aldao et al., 2010) and dissociation (Tibubos et al., 2018). Meanwhile, in adolescents it is associated with superior emotional control (Lantrip et al., 2016). Thus, reduced cognitive reappraisal may plausibly be associated with greater dissociation and panic symptoms–a possibility particularly relevant to adolescents, who are less adept at emotion regulation than adults given their stage of neural development (Casey et al., 2008; Hare et al., 2008). It is noted that cognitive *re*appraisal is distinct from the cognitive appraisals of dissociation construct described above. The latter refers to the content of the appraisal, or the beliefs that the individual holds about their dissociative experience (Černis et al., 2020), whereas the former is an emotion regulation strategy in which experiences are reinterpreted in a way that reduces their level of perceived threat (Gross & John, 2003).

Finally, alexithymia (characterised by difficulty identifying and describing emotional states, alongside a tendency to focus attention externally; Bagby et al., 1994) may also mediate the relationship between dissociation and panic symptoms. There are higher levels of alexithymia amongst adults with panic disorder (27%; Šago et al., 2020) than in the general population (10%; Franz et al., 2008), and difficulty identifying feelings is positively associated with depersonalisation or derealisation in this clinical group (Majohr et al., 2011). In adolescents, there is a similar association between alexithymia and dissociative experiences (Kekkonen et al., 2021). Thus, it is possible that individuals with alexithymia experience dissociative symptoms with greater intensity and catastrophically misinterpret them (Hunter et al., 2003), thus triggering a panic response. This is particularly likely considering the relationships between difficulty identifying and describing feelings, sensory amplification (the tendency to experience a typical physical sensation as intense), and a fear of body sensations associated with autonomic arousal (De Berardis et al., 2007). However, of note, a recent network analysis by Černis, Ehlers, and Freeman (2022) found that higher levels of dissociation related to lower levels of alexithymia in adults. This was interpreted to indicate that individuals who experience FSA‐dissociation self‐report an increased ability to notice their emotions. Such heightened sensitivity to affect could be explained as hyper‐vigilance to threat, in light of co‐occurrent affect intolerance. It is important to assess the role of alexithymia given these inconsistencies.

This study assesses the longitudinal relationships between panic symptoms and dissociation in adolescents, and potential mediating variables. Our hypotheses (see Figure S1 for an outline of hypothesised associations) were as follows:

1. There will be a significant longitudinal relationship between dissociation (focusing on the FSA‐dissociation subtype) at Time 1 and symptoms of panic disorder 1 month later, at Time 2, such that more severe dissociation will be related to more severe panic symptoms.
2. The relationship between dissociation and panic disorder symptoms will be mediated by (a) cognitive appraisals of dissociation, (b) emotion regulation strategies (expressive suppression and cognitive reappraisal), and (c) alexithymia.

## METHOD

### Participants

Adolescents were eligible to participate if they were aged 13–18 years, lived in the UK, and had no identified learning disability. See Table 1 for demographic information and Figure S2 for participant recruitment and flow through the study.

**TABLE 1:** Participants' demographic information.

	*n* = 421
Age (years)	
Mean (SD, range)	16.71 (SD = 1.37, 13–18)
Gender, *n* (%)	
Male	55 (13.10%)
Female	292 (69.40%)
Other	62 (14.70%)
Prefer not to say	12 (2.90%)
Ethnicity, *n* (%)	
Any White background	366 (86.9%)
Any mixed background	19 (4.50%)
Any Asian background	22 (5.20%)
Any black background	4 (0.90%)
Any other ethnic group	9 (2.10%)
Prefer not to state ethnicity	1 (0.20%)
Socioeconomic status, *n* (%)	
Professional	264 (62.7%)
Other employed	109 (25.9%)
Unemployed, student, or retired	37 (8.8%)
Unknown	11 (2.6%)

The mean age of participants was 16.71 years (SD = 1.37), and 69.4% reported their gender as ‘female’. 62.7% reported having at least one parent with a professional occupation, and 86.9% reported their ethnicity as White.

Eight thousand four hundred thirty‐six young people opened the survey link at the first time point, and 4501 (53.4%) gave informed assent/consent (and parent consent where applicable). These 4501 individuals were emailed the link to the second survey 1 month later, and 421 (9.4% of those sent the link) provided adequate data on all measures at this stage.

### Design

Participants completed the same battery of questionnaires (Panic Disorder Severity Scale for Children and Adolescents, Černis Felt Sense of Anomaly, Emotion Regulation Questionnaire for Children and Adolescents, Alexithymia Questionnaire for Children, and Cognitive Appraisals of Dissociation in Psychosis) at two time points 1 month apart. Ethical approval was granted by the University of Oxford Medical Sciences Interdivisional Research Ethics Committee (reference: R77368/RE001). The study was pre‐registered on the Open Science Framework (https://osf.io/jd973/).

Participants were recruited in November 2021 via advertisements on Twitter, Facebook, and Instagram. Adverts included a link to a Qualtrics landing page, where information about the study was presented. Informed consent/assent was provided, followed by answers to the first set of surveys. One month later, participants (or their parent/guardian if aged 13–15 years) were emailed the link to the second survey. On its completion, they were offered the chance to enter a draw to win a gift voucher (see Figure S3 for procedure outline).

### Power analysis

An a priori power analysis, conducted with G*Power v.3 (Faul et al., 2009), found that a sample size of 68 would be required to find a medium effect size with power (1 – β) = .80 and *α* = .05. Allowing for a drop‐out rate of 50% at Time 2 (based on a previous longitudinal study of anxiety in adolescents; Damian et al., 2017) resulted in a suggested sample size of 136 participants at Time 1. The final number of recruited participants far exceeded this, resulting in a highly powered study.

### Measures

#### Preliminary consultation with a young person

Prior to the study, a young person (aged 14 years) was consulted to ensure the suitability of the questionnaires for adolescents. Subsequently, minor changes were made: “hot flushes” was clarified in the Panic Disorder Severity Scale for Children and Adolescents with the explanation “suddenly feeling very hot”.

#### Demographic information

Participants provided demographic information (age, gender, ethnic background, and parent/guardian occupation) at Time 1. Socioeconomic status (SES) was coded from parent/guardian occupations according to the Office for National Statistics Standard Occupation Classification 2010 (ONS, 2010). Codes were subsequently collapsed into four groups (‘professional’, ‘other employed’, ‘unemployed, student, or retired’, and ‘unknown’). Where two parent/guardian occupations were provided, the highest SES category was used.

#### Panic symptoms measure

The Panic Disorder Severity Scale for Children and Adolescents (PDSS‐A; Pincus et al., 2008) is an adaptation of the Panic Disorder Severity Scale (Shear et al., 1997) suitable for adolescents aged 11–17 years. This 7‐item self‐report measure assesses panic frequency, panic‐related distress, anticipatory anxiety, agoraphobia, avoidance, fear associated with physical symptoms of panic, and related impairments. It uses a 5‐point Likert scale for each item (e.g., ‘How many panic and limited symptom attacks did you have during the past week?’). Scores are summed to yield a total within the range 0–28, with higher scores indicating greater severity. The PDSS‐A has acceptable internal consistency (*α* = .82) and 1‐day test‐retest reliability (*r* = .79; Elkins et al., 2014). In this study, it had excellent internal reliability (Cronbach's *α* = .91).

#### Dissociation measure

The Černis Felt Sense of Anomaly (ČEFSA; Černis et al., 2021) questionnaire is a 35‐item self‐report measure assessing the frequency of experiences characterised by FSA‐dissociation over the past two weeks. It consists of 7 factors: Anomalous Experience of the Self, Anomalous Experience of the Physical Body, Altered Sense of Familiarity, Anomalous Experience of Emotion, Altered Sense of Connection, Altered Sense of Agency, and Altered Sense of Reality. Each item (e.g., ‘I feel like a stranger to myself’) is rated from 0 (‘never’) to 4 (‘always’), and responses are summed to give a total in the range 0–140. The ČEFSA has good internal consistency (*α* = .86–.92) in adult non‐clinical (Černis et al., 2021) and adolescent community samples (Cronbach's *α* = .97; Lofthouse et al., 2023). In this study, it had excellent internal consistency (Cronbach's *α* = .97).

#### Emotion regulation measure

The Emotion Regulation Questionnaire for Children and Adolescents (ERQ‐CA; Gullone & Taffe, 2012) is an adaptation of the Emotion Regulation Questionnaire (Gross & John, 2003). Items are rated on a 5‐point scale (1 = ‘strongly disagree’, 5 = ‘strongly agree’). The measure has two subscales: cognitive reappraisal (e.g., ‘When I'm worried about something, I make myself think about it in a way that helps me feel better’), and expressive suppression (e.g., ‘I keep my feelings to myself’). Total score ranges between 6–30 for cognitive reappraisal and 4–20 for expressive suppression. Both subscales have demonstrated good internal consistency and stable intraclass correlation coefficients over a 12‐month period in a sample of young people aged 10–18 years (Gullone & Taffe, 2012). In this study, they had acceptable internal consistencies (Cronbach's *α* = .73 and *α* = .75 for expressive suppression and cognitive reappraisal subscales, respectively).

#### Alexithymia measure

Alexithymia was evaluated using two subscales from the Alexithymia Questionnaire for Children (AQC; Rieffe et al., 2006): Difficulty Identifying Feelings and Difficulty Describing Feelings. Although there is a third subscale (Externally‐Oriented Thinking) in the AQC, Loas et al. (2017) found that it has low reliability in adolescent populations, and therefore recommended that it is excluded. Instead, the Difficulty Identifying Feelings and Difficulty Describing Feelings subscales are totalled to give a single alexithymia score. These subscales together comprise 12 items, rated on a scale from 0 (‘not true’) to 2 (‘true’), with a maximum score of 24. Loas et al. (2017) found that this 12‐item measure had good psychometric properties in an adolescent population (Cronbach's *α* = .83); this was replicated in the current study (Cronbach's *α* = .78).

#### Cognitive appraisals of dissociation measure

The Cognitive Appraisals of Dissociation in Psychosis (CAD‐P; Černis et al., 2020) measure is a 13‐item self‐report scale assessing catastrophic cognitive appraisals of dissociation. Appraisals reflect concerns about dissociative experiences being inherently dangerous or signs of threat (e.g., ‘I'm losing my mind’), negative beliefs about the self (e.g., ‘I am all alone’) and fears about control and ownership over oneself and one's actions (‘It's not me in control right now’). Although originally developed for psychosis, items reflect appraisals that are common in the context of anxiety. Items are rated on a 5‐point scale from ‘never’ to ‘always’, indicating how often these statements come to mind when participants are ‘feeling strange, disconnected, unreal, or dissociated’. All items load onto one factor, resulting in a total within the range 0–52. Higher scores indicate greater concern that experiences are dangerous or indicate impending threat. The CAD‐P has good test re‐test reliability and internal consistency in adult clinical and non‐clinical samples (Černis et al., 2020), and an adolescent community sample (Cronbach's *α* = .92; Lofthouse et al., 2023). It had excellent internal consistency in this study (Cronbach's *α* = .92).

### Ethical considerations

Adolescents aged 16–18 years provided informed consent, whereas those aged 13–15 years provided informed assent and their parent/guardian provided informed consent.

### Data analysis

Data were analysed using SPSS v.28.0 (IBM Corp, 2021), and the PROCESS v.4.0 package, Model 4 (Hayes, 2013). Data were included if at least 80% of items were answered for each measure, and it was clear that responses had been given in an appropriate manner (e.g., not all maximum/minimum choices selected for every question of every questionnaire). Included data had very low levels of missing values (0.0004%); these were deleted listwise. Preliminary data screening indicated deviations from normal distribution (according to Shapiro‐Wilk and Kolmogorov‐Smirnov values). However, given the power afforded by the large sample, use of parametric tests was warranted and therefore Pearson's correlation coefficients were calculated (Ghasemi & Zahediasl, 2012).

The necessary assumptions for mediation analysis were met, with the exception of homoscedasticity. There were linear relationships between variables and Durbin‐Watson statistics indicated that observations were independent. There was an absence of multicollinearity as shown by variance inflation factor and tolerance values, and residuals were normally distributed. However, given the small degree of heteroscedasticity (demonstrated in the scatterplots of the standardised residuals against the values of the outcome variables), bootstrapping was used as this technique does not require standard errors and thus is more robust (Hayes & Scharkow, 2013). 95% confidence intervals were generated using 5000 bootstrap samples. Regression analyses were conducted to reveal relationships between variables, and the total, direct, and indirect effects of the independent variable on the dependent variable were used to assess whether mediation was supported (Preacher & Hayes, 2004). All reported coefficients are standardised (unless stated otherwise). *α* = .05 two‐tailed was the criterion for statistical significance. Finally, we conducted an exploratory analysis of the relationship between the variables controlling for baseline panic symptoms, by adding PDSS‐A score at Time 1 as a covariate (Loh & Ren, 2023).

## RESULTS

Table 2 provides the mean, standard deviation, and range for each measure.

**TABLE 2:** Descriptive statistics for all measures (*n* = 421).

	Mean	Std. deviation	Scale min‐max	Observed range
Felt sense of anomaly dissociation (FSA‐dissociation; time 1)	65.45	31.72	0–140	0–134
Cognitive reappraisal (ERQ‐CA; time 1)	17.47	4.04	6–30	7–30
Expressive suppression (ERQ‐CA; time 1)	13.78	3.37	4–20	4–20
Alexithymia (AQC; time 1)	17.18	4.85	0–24	1–24
Cognitive appraisals of dissociation (CAD‐P; time 1)	26.68	12.06	0–52	0–52
Panic symptoms (PDSS‐A; time 1)	9.28	6.24	0–28	0–26
Felt sense of anomaly dissociation (FSA‐dissociation; time 2)	64.80	33.87	0–140	0–140
Cognitive reappraisal (ERQ‐CA; time 2)	17.71	4.55	6–30	6–30
Expressive suppression (ERQ‐CA; time 2)	13.90	3.35	4–20	4–20
Alexithymia (AQC; time 2)	17.16	5.14	0–24	0–24
Cognitive appraisals of dissociation (CAD‐P; time 2)	26.11	12.83	0–52	0–52
Panic symptoms (PDSS‐A; time 2)	9.19	6.15	0–28	0–25

Table 3 provides Pearson's correlation co‐efficients. All variables were significantly correlated in the predicted directions.

**TABLE 3:** Correlation matrix.

	Panic symptoms time 1	Cognitive reappraisal time 1	Expressive suppression time 1	Alexithymia time 1	Cognitive appraisals of dissociation time 1	Felt sense of anomaly time 1	Cognitive reappraisal time 2	Expressive suppression time 2	Panic symptoms time 2	Cognitive appraisals of dissociation time 2	Felt sense of anomaly time 2	Alexithymia time 2
Panic symptoms time 1												
Pearson correlation	1	−.209**	.280**	.502**	.626**	.597**	−.210**	.302**	.805**	.560**	.581**	.493**
Sig. (2‐tailed)		<.001	<.001	<.001	<.001	<.001	<.001	<.001	<.001	<.001	<.001	<.001
Cognitive reappraisal time 1												
Pearson correlation	−.209**	1	−.052	−.219**	−.210**	−.191**	.611**	−.036	−.211**	−.153**	−.192**	−.223**
Sig. (2‐tailed)	<.001		.286	<.001	<.001	<.001	<.001	.456	<.001	.002	<.001	<.001
Expressive suppression time 1												
Pearson correlation	.280**	−.052	1	.480**	.339**	.332**	−.05	.747**	.296**	.284**	.347**	.427**
Sig. (2‐tailed)	<.001	.286		<.001	<.001	<.001	.304	<.001	<.001	<.001	<.001	<.001
Alexithymia time 1												
Pearson correlation	.502**	−.219**	.480**	1	.623**	.631**	−.179**	.499**	.469**	.551**	.582**	.813**
Sig. (2‐tailed)	<.001	<.001	<.001		<.001	<.001	<.001	<.001	<.001	<.001	<.001	<.001
Cognitive appraisals of dissociation time 1												
Pearson correlation	.626**	−.210**	.339**	.623**	1	.820**	−.202**	.353**	.599**	.820**	.753**	.572**
Sig. (2‐tailed)	<.001	<.001	<.001	<.001		<.001	<.001	<.001	<.001	<.001	<.001	<.001
Felt sense of anomaly time 1												
Pearson correlation	.597**	−.191**	.332**	.631**	.820**	1	−.138**	.367**	.583**	.780**	.881**	.597**
Sig. (2‐tailed)	<.001	<.001	<.001	<.001	<.001		.005	<.001	<.001	<.001	<.001	<.001
Cognitive reappraisal time 2												
Pearson correlation	−.210**	.611**	−.05	−.179**	−.202**	−.138**	1	.021	−.204**	−.123*	−.138**	−.132**
Sig. (2‐tailed)	<.001	<.001	.304	<.001	<.001	.005		.669	<.001	.012	.004	.007
Expressive suppression time 2												
Pearson correlation	.302**	−.036	.747**	.499**	.353**	.367**	.021	1	.337**	.382**	.418**	.494**
Sig. (2‐tailed)	<.001	.456	<.001	<.001	<.001	<.001	.669		<.001	<.001	<.001	<.001
Panic symptoms time 2												
Pearson correlation	.805**	−.211**	.296**	.469**	.599**	.583**	−.204**	.337**	1	.588**	.611**	.509**
Sig. (2‐tailed)	<.001	<.001	<.001	<.001	<.001	<.001	<.001	<.001		<.001	<.001	<.001
Cognitive appraisals of dissociation time 2												
Pearson correlation	.560**	−.153**	.284**	.551**	.820**	.780**	−.123*	382**	.588**	1	.867**	.634**
Sig. (2‐tailed)	<.001	.002	<.001	<.001	<.001	<.001	.012	<.001	<.001		<.001	<.001
Felt sense of anomaly time 2												
Pearson correlation	.581**	−.192**	.347**	.582**	.753**	.881**	−.138**	.418**	.611**	.867**	1	.655**
Sig. (2‐tailed)	<.001	<.001	<.001	<.001	<.001	<.001	.004	<.001	<.001	<.001		<.001
Alexithymia time 2												
Pearson correlation	.493**	−.223**	.427**	.813**	.572**	.597**	−.132**	.494**	.509**	.634**	.655**	1
Sig. (2‐tailed)	<.001	<.001	<.001	<.001	<.001	<.001	.007	<.001	<.001	<.001	<.001	

*Correlation is significant at the .05 level (2‐tailed).

**Correlation is significant at the .01 level (2‐tailed).

### Longitudinal relationship between FSA‐dissociation and panic symptoms

FSA‐dissociation (Time 1) was positively associated with panic symptoms (Time 2; β = .24, SE = 0.013, *p* < .001).

### Mediation of the relationship between FSA‐dissociation and panic symptoms

**FIGURE 1:**
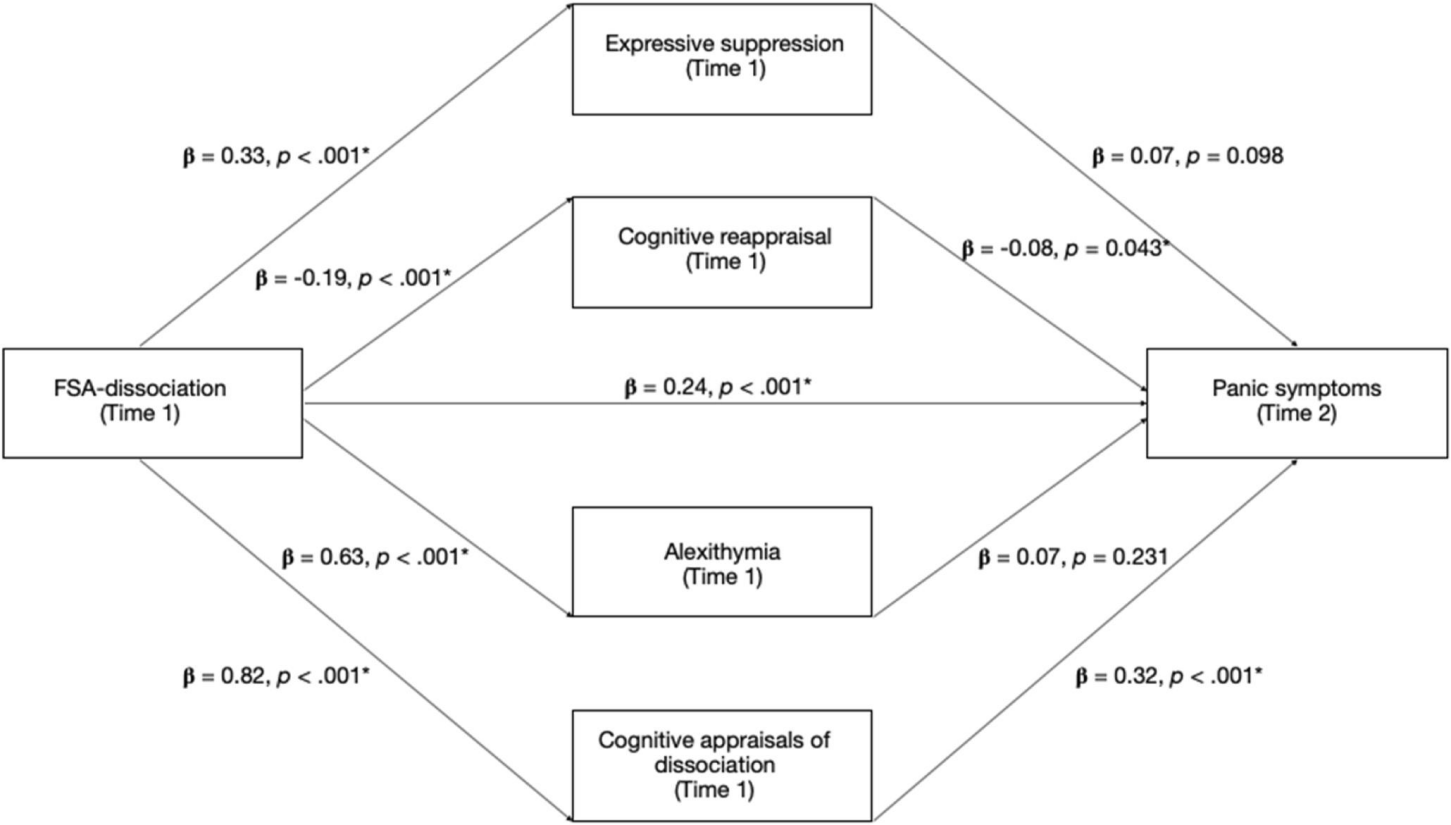
The relationship between felt sense of anomaly dissociative experiences and panic symptoms and the mediating roles of expressive suppression, cognitive reappraisal, alexithymia and cognitive appraisals of dissociation. All reported coefficients are standardised. Significance at *p* < .05.

The relationship between FSA‐dissociation and panic symptoms, and its mediators, are presented in Figure 1. Statistics are presented in Table 4. The relationship between FSA‐dissociation (Time 1) and panic symptoms (Time 2) was significantly mediated by cognitive appraisals of dissociation (indirect effect size = 0.0510, bootstrapped SE = 0.0120, bootstrapped 95% CI = 0.0277–0.0747). Higher levels of FSA‐dissociation were associated with more negative cognitive appraisals of dissociation (β = .82, SE = 0.0106, *p* < .001), which in turn associated with more severe panic symptoms (β = .32, SE = 0.0350, *p* < .001).

**TABLE 4:** FSA‐dissociation regression and mediation analyses.

Regression analysis	β	Standard error	*t*	*p*	95% confidence interval	
					Lower	Upper
FSA‐dissociation on expressive suppression	.33	0.005	7.20	<.001	0.026	0.045
FSA‐dissociation on cognitive reappraisal	−.19	0.006	−3.99	<.001	−0.036	−0.012
FSA‐dissociation on alexithymia	.63	0.006	16.65	<.001	0.085	0.108
FSA‐dissociation on cognitive appraisals of dissociation	.82	0.011	29.32	<.001	0.291	0.333
Expressive suppression on panic symptoms	.07	0.080	1.66	.098	−0.025	0.288
Cognitive reappraisal on panic symptoms	−.08	0.060	−2.03	.043	−0.239	−0.004
Alexithymia on panic symptoms	.07	0.069	1.20	.231	−0.053	0.218
Cognitive appraisals of dissociation on panic symptoms	.32	0.035	4.67	<.001	0.095	0.232
FSA‐dissociation on panic symptoms	.24	0.013	3.49	<.001	0.020	0.073

Mediation analysis	Effect	Standard error	*t*	*p*	95% confidence interval	
					Lower	Upper
Total effect of FSA‐dissociation on panic symptoms	0.11	0.008	14.7017	<.001	0.098	0.128
Direct effect of FSA‐dissociation on panic symptoms	0.05	0.013	3.4889	<.001	0.020	0.073

Indirect effects of FSA‐Dissociation on panic symptoms	Effect	Bootstrapped standard error	Bootstrapped 95% confidence interval	
			Lower	Upper
Via expressive suppression	0.005	0.003	−0.005	0.011
Via cognitive reappraisal	0.003	0.002	0.0001	0.007
Via alexithymia	0.008	0.006	−0.005	0.021
Via cognitive appraisals of dissociation	0.051	0.012	0.028	0.075

The emotion regulation strategy of cognitive reappraisal was also a significant mediating variable (indirect effect size = 0.0030, bootstrapped SE = 0.0017, bootstrapped 95% CI = 0.0001–0.0068), such that higher levels of FSA‐dissociation were associated with greater cognitive reappraisal (β = −.19, SE = 0.0061, *p* < .001). In turn, higher levels of cognitive reappraisal were associated with less severe panic symptoms (β = −.08, SE = 0.0598, *p* = .043). These mediation effects were partial, given the significant direct effect of FSA‐dissociation on panic symptoms (direct effect size = 0.0466, SE = 0.0134, *p* < .001, bootstrapped 95% CI = 0.020–0.073).

Neither expressive suppression (indirect effect size = 0.005, bootstrapped SE = 0.003, bootstrapped 95% CI = −0.005–0.011) nor alexithymia (indirect effect size = 0.008, bootstrapped SE = 0.006, bootstrapped 95% CI = 0.005–0.021) were significant mediators.

### Mediation of the relationship between FSA‐dissociation and panic symptoms controlling for baseline panic symptoms

When PDSS‐A score at Time 1 was added as a covariate to control for panic symptoms at baseline, the partial mediating effects of both cognitive appraisals of dissociation (indirect effect size = 0.0098, bootstrapped SE = 0.0082, bootstrapped 95% CI = −0.0063–0.0259) and cognitive reappraisal (indirect effect size = 0.0007, bootstrapped SE = 0.0008, bootstrapped 95% CI = −0.0005–0.0025) failed to retain their significance. See Table S1 for full results.

## DISCUSSION

This study explores the effects of cognitive appraisals of dissociation, emotion regulation strategies (expressive suppression and cognitive reappraisal) and alexithymia on the relationship between dissociative experiences (focusing on the FSA‐dissociation subtype) and panic symptoms within a community adolescent sample. As hypothesised, we found significant longitudinal relationships between felt sense of anomaly dissociative experiences and panic symptoms. The second hypothesis regarding mediation was partly supported; the emotion regulation strategy of cognitive reappraisal and cognitive appraisals of the dissociative experience (but not alexithymia nor expressive suppression) partially mediated the relationship between FSA‐dissociation and panic. However, exploratory analyses showed that this effect failed to retain its significance when controlling for panic symptoms at baseline.

The positive relationship between FSA‐dissociation and panic symptoms aligns with reported associations between these two experiences in adult (Cassano et al., 1989; Marshall et al., 2000; Seguí et al., 2000) and adolescent populations (Doerfler et al., 2007). Under a cognitive‐behavioural interpretation, this finding may indicate that the presence and misinterpretation of dissociative experiences can result in escalating anxiety, heightened dissociation, and panic symptoms (Clark, 1986; Hunter et al., 2003).

Cognitive appraisals of dissociation was a partial mediator of the longitudinal relationship between dissociation and panic symptoms at Time 2. Greater levels of dissociation at the first time point were correlated with more catastrophic appraisals (e.g., ‘I can't trust my own mind’), which in turn were positively associated with more severe panic symptoms. These results align with models proposed by Clark (1986; panic), Hunter et al. (2003; depersonalisation), and Černis et al. (2022a; FSA‐dissociation), which highlight the role of negative appraisals in the maintenance of psychopathological symptoms. For example, at the centre of Clark's cognitive model of panic (1986) is the misinterpretation of internal sensations as inherently dangerous. However, it is noted that the measure of cognitive appraisals of dissociation used here also included items relating to negative beliefs about oneself (e.g., ‘I am all alone’), which do not typically feature in models. Negative self‐beliefs have been identified in qualitative accounts of panic in this age group (Baker et al., 2022; Hewitt et al., 2021) and may be particularly relevant for adolescents during this period of identity development (Erikson, 1968).

Catastrophic interpretations are maintained when individuals fail to re‐evaluate dissociative symptoms in a less threatening manner; this was demonstrated by the partial mediating effect of the emotion regulation strategy of cognitive reappraisal. Greater dissociative symptoms at the first time point related to less cognitive reappraisal, which in turn was associated with more severe panic symptoms at the second time point. Thus, when individuals prone to dissociation fail to reappraise their symptoms in a way that reduces their negative impact, the resulting escalation of anxiety may heighten the feared dissociation sensation (Hunter et al., 2003; Černis et al., 2022a, 2022b) and panic symptoms.

Notably, however, the mediating effects of cognitive appraisals of dissociation and cognitive reappraisal were no longer significant when baseline panic symptoms were controlled for in the analyses. This may be explained by the stability of symptoms across the two time points (evidenced by the strong correlation between Time 1 and Time 2 PDSS‐A scores; *r* > .80). This stability, potentially due to the relatively short time lag between assessments, means that although our results provide important insight into the nature of the links between dissociation and panic symptoms, they cannot speak to their temporal relationships. Given this lack of variance in panic symptoms across timepoints, our findings raise the possibility that the relationship between FSA‐dissociation and panic is bidirectional, with each influencing the other in a more cyclical manner than originally hypothesised. It is both plausible and consistent with a cognitive‐behavioural interpretation that dissociation can trigger panic symptoms–but equally, panic symptoms themselves could lead to dissociative experiences. The strong correlations between variables provide preliminary evidence for this interpretation. However, experimental evidence is needed to confirm whether the combination of catastrophic misinterpretations of dissociation and a lack of reappraisal is not only co‐morbid with panic symptoms but plays a causal role in their occurrence. Future studies with longer durations between time points and at least three assessments might expect to find greater indirect effects and would provide additional insight into temporal precedence.

Neither alexithymia nor expressive suppression significantly mediated the longitudinal relationship between dissociative experiences and panic symptoms. However, expressive suppression was significantly related to greater levels of FSA‐dissociation. Thus, adolescents who experience greater dissociation are perhaps more likely to regulate their emotions through suppressive behaviours. However, since the measures of dissociative experiences and emotion regulation used in the mediation analyses were recorded at the same point in time, the direction of this relationship is unclear. Furthermore, the main analysis found that expressive suppression reported at the first time point was not significantly related to panic symptoms reported 1 month later. This may reflect the importance of hyper‐vigilance to physiological changes in the development and maintenance of panic symptoms (Clark, 1986)–something one could logically expect to be absent in the context of expressive suppression, where affect‐related symptoms are purposefully ignored or avoided.

A key strength of this study is its large community sample; however, as outlined above, future research would benefit from including a greater number of assessment stages with longer time lags to provide richer insights into temporal precedence. Further studies should also aim to replicate these results in adolescents with panic disorder, who may experience qualitatively different panic and dissociation symptoms. Additionally, it would be beneficial to include more diverse samples with respect to gender, socio‐economic status, and ethnicity and assess a greater number of emotion regulation strategies. Cognitive reappraisal and expressive suppression are just two techniques from a wide repertoire, and clinical groups might utilise different strategies to non‐patients—for instance, rumination or catastrophizing (Garnefski et al., 2002).

## CONCLUSION

This study found a significant positive relationship between dissociative experiences and panic symptoms reported 1 month later in a large adolescent sample. This was mediated by the emotion regulation strategy of cognitive reappraisal, and cognitive appraisals of dissociation; although these mediation effects failed to retain their significance when controlling for initial panic symptoms, this is likely due to the stability of panic symptoms across both assessment points. However, the pattern of results is consistent with existing models (Clark, 1986; Hunter et al., 2003), in which dissociative experiences, when misinterpreted in a catastrophic manner, may lead to escalating panic symptoms. This could maintain or heighten the feared dissociation sensation. Further research is needed to explore temporal and causal relationships between these variables.

## AUTHOR CONTRIBUTIONS

**Lottie Shipp**: Conceptualization; data curation; formal analysis; investigation; methodology; writing – original draft; writing – review & editing. **Alisa Musatova**: Conceptualization; data curation; investigation; methodology; writing – original draft. **Emma Černis**: Conceptualization; methodology; project administration; supervision; writing – original draft; writing – review & editing. **Polly Waite**: Conceptualization; methodology; project administration; supervision; writing – original draft; writing – review & editing.

## CONFLICT OF INTEREST STATEMENT

The authors have declared that they have no competing or potential conflicts of interest.

## ETHICAL CONSIDERATIONS

Ethical approval was granted by the University of Oxford Medical Sciences Interdivisional Research Ethics Committee (reference: R77368/RE001).

## Supporting information

Supporting Information S1

## Data Availability

The data that support the findings of this study are available on request from the corresponding author. The data are not publicly available due to privacy or ethical restrictions.

## References

[jcv212202-bib-0001] Aafjes‐van Doorn, K. , Zilcha‐Mano, S. , Graham, K. , Caldari, A. , Barber, J. P. , Chambless, D. L. , & Milrod, B. (2019). The role of safety behaviors in panic disorder treatment: Self‐regulation or self‐defeat? Journal of Contemporary Psychotherapy, 49(4), 203–212. 10.1007/s10879-019-09432-9 PMC7678808 33223564

[jcv212202-bib-0002] Achiam‐Montal, M. , Tibi, L. , & Lipsitz, J. D. (2013). Panic disorder in children and adolescents with noncardiac chest pain. Child Psychiatry and Human Development, 44(6), 742–750. 10.1007/s10578-013-0367-9 23378228

[jcv212202-bib-0003] Aldao, A. , Nolen‐Hoeksema, S. , & Schweizer, S. (2010). Emotion‐regulation strategies across psychopathology: A meta‐analytic review. Clinical Psychology Review, 30(2), 217–237. 10.1016/j.cpr.2009.11.004 20015584

[jcv212202-bib-0004] Bagby, R. M. , Parker, J. D. A. , & Taylor, G. J. (1994). The twenty‐item Toronto alexithymia scale—I. Item selection and cross‐validation of the factor structure. Journal of Psychosomatic Research, 38(1), 23–32. 10.1016/0022-3999(94)90005-1 8126686

[jcv212202-bib-0005] Baker, H. J. , Hollywood, A. , & Waite, P. (2022). Adolescents’ lived experience of panic disorder: An interpretative phenomenological analysis. BMC Psychology, 10(1), 143. 10.1186/s40359-022-00849-x 35668509 PMC9167912

[jcv212202-bib-0006] Baker, R. , Holloway, J. , Thomas, P. W. , Thomas, S. , & Owens, M. (2004). Emotional processing and panic. Behaviour Research and Therapy, 42(11), 1271–1287. 10.1016/j.brat.2003.09.002 15381438

[jcv212202-bib-0007] Casey, B. J. , Getz, S. , & Galvan, A. (2008). The adolescent brain. Developmental Review, 28(1), 62–77. 10.1016/j.dr.2007.08.003 18688292 PMC2500212

[jcv212202-bib-0008] Cassano, G. B. , Petracca, A. , Perugi, G. , Toni, C. , Tundo, A. , & Roth, M. (1989). Derealization and panic attacks: A clinical evaluation on 150 patients with panic disorder/agoraphobia. Comprehensive Psychiatry, 30(1), 5–12. 10.1016/0010-440x(89)90112-0 2924566

[jcv212202-bib-0009] Cavicchioli, M. , Scalabrini, A. , Northoff, G. , Mucci, C. , Ogliari, A. , & Maffei, C. (2021). Dissociation and emotion regulation strategies: A meta‐analytic review. Journal of Psychiatric Research, 143, 370–387. 10.1016/j.jpsychires.2021.09.011 34592484

[jcv212202-bib-0010] Černis, E. , Beierl, E. , Molodynski, A. , Ehlers, A. , & Freeman, D. (2021). A new perspective and assessment measure for common dissociative experiences: ‘Felt sense of anomaly’. PLoS One, 16(2), e0247037. 10.1371/journal.pone.0247037 33626089 PMC7904139

[jcv212202-bib-0011] Černis, E. , Bird, J. C. , Molodynski, A. , Ehlers, A. , & Freeman, D. (2020). Cognitive appraisals of dissociation in psychosis: A new brief measure. Behavioural and Cognitive Psychotherapy, 49(4), 472–484. 10.1017/S1352465820000958 PMC8293624 33446299

[jcv212202-bib-0012] Černis, E. , Ehlers, A. , & Freeman, D. (2022a). Psychological mechanisms connected to dissociation: Generating hypotheses using network analyses. Journal of Psychiatric Research, 148, 165–173. 10.1016/j.jpsychires.2022.01.049 35124396 PMC8968218

[jcv212202-bib-0013] Černis, E. , Molodynski, A. , Ehlers, A. , & Freeman, D. (2022b). Dissociation in patients with non‐affective psychosis: Prevalence, symptom associations, and maintenance factors. Schizophrenia Research, 239, 11–18. 10.1016/j.schres.2021.11.008 34800911 PMC8765411

[jcv212202-bib-0014] Clark, D. M. (1986). A cognitive approach to panic. Behaviour Research and Therapy, 24(4), 461–470. 10.1016/0005-7967(86)90011-2 3741311

[jcv212202-bib-0015] Clark, D. M. (1999). Anxiety disorders: Why they persist and how to treat them. Behaviour Research and Therapy, 37, S5–S27. 10.1016/S0005-7967(99)00048-0 10402694

[jcv212202-bib-0016] Damian, L. E. , Negru‐Subtirica, O. , Stoeber, J. , & Băban, A. (2017). Perfectionistic concerns predict increases in adolescents’ anxiety symptoms: A three‐wave longitudinal study. Anxiety, Stress & Coping, 30(5), 551–561. 10.1080/10615806.2016.1271877 27966368

[jcv212202-bib-0017] De Berardis, D. , Campanella, D. , Gambi, F. , La Rovere, R. , Sepede, G. , Core, L. , Canfora, G. , Santilli, E. , Valchera, A. , Mancini, E. , Salerno, R. M. , Moschetta, F. S. , & Ferro, F. M. (2007). Alexithymia, fear of bodily sensations, and somatosensory amplification in young outpatients with panic disorder. Psychosomatics, 48(3), 239–246. 10.1176/appi.psy.48.3.239 17478593

[jcv212202-bib-0018] Doerfler, L. A. , Connor, D. F. , Volungis, A. M. , & Toscano, P. F. (2007). Panic disorder in clinically referred children and adolescents. Child Psychiatry and Human Development, 38(1), 57–71. 10.1007/s10578-006-0042-5 17186364

[jcv212202-bib-0019] Elkins, R. M. , Pincus, D. B. , & Comer, J. S. (2014). A psychometric evaluation of the panic disorder severity scale for children and adolescents. Psychological Assessment, 26(2), 609–618. 10.1037/a0035283 24295237 PMC4049332

[jcv212202-bib-0020] Erikson, E. H. (1968). Identity: Youth and crisis. W.W. Norton.

[jcv212202-bib-0021] Faul, F. , Erdfelder, E. , Buchner, A. , & Lang, A.‐G. (2009). Statistical power analyses using G*Power 3.1: Tests for correlation and regression analyses. Behavior Research Methods, 41(4), 1149–1160. 10.3758/BRM.41.4.1149 19897823

[jcv212202-bib-0022] Franz, M. , Popp, K. , Schaefer, R. , Sitte, W. , Schneider, C. , Hardt, J. , Decker, O. , & Braehler, E. (2008). Alexithymia in the German general population. Social Psychiatry and Psychiatric Epidemiology, 43(1), 54–62. 10.1007/s00127-007-0265-1 17934682

[jcv212202-bib-0023] Garnefski, N. , Legerstee, J. , Kraaij, V. , Van Den Kommer, T. , & Teerds, J. (2002). Cognitive coping strategies and symptoms of depression and anxiety: A comparison between adolescents and adults. Journal of Adolescence, 25(6), 603–611. 10.1006/jado.2002.0507 12490178

[jcv212202-bib-0024] Ghasemi, A. , & Zahediasl, S. (2012). Normality tests for statistical analysis: A guide for non‐statisticians. International Journal of Endocrinology and Metabolism, 10(2), 486–489. 10.5812/ijem.3505 23843808 PMC3693611

[jcv212202-bib-0025] Gross, J. J. (1998). The emerging field of emotion regulation: An integrative review. Review of General Psychology, 2(3), 271–299. 10.1037/1089-2680.2.3.271

[jcv212202-bib-0026] Gross, J. J. (2002). Emotion regulation: Affective, cognitive, and social consequences. Psychophysiology, 39(3), 281–291. 10.1017/s0048577201393198 12212647

[jcv212202-bib-0027] Gross, J. J. , & John, O. P. (2003). Individual differences in two emotion regulation processes: Implications for affect, relationships, and well‐being. Journal of Personality and Social Psychology, 85(2), 348–362. 10.1037/0022-3514.85.2.348 12916575

[jcv212202-bib-0028] Gullone, E. , & Taffe, J. (2012). The emotion regulation questionnaire for children and adolescents (ERQ–CA): A psychometric evaluation. Psychological Assessment, 24(2), 409–417. 10.1037/a0025777 22023559

[jcv212202-bib-0029] Hare, T. A. , Tottenham, N. , Galvan, A. , Voss, H. U. , Glover, G. H. , & Casey, B. J. (2008). Biological substrates of emotional reactivity and regulation in adolescence during an emotional go‐nogo task. Biological Psychiatry, 63(10), 927–934. 10.1016/j.biopsych.2008.03.015 18452757 PMC2664095

[jcv212202-bib-0030] Hayes, A. (2013). Introduction to mediation, moderation, and conditional process analysis: A regression‐based approach. Guildford Publications.

[jcv212202-bib-0031] Hayes, A. F. , & Scharkow, M. (2013). The relative trustworthiness of inferential tests of the indirect effect in statistical mediation analysis: Does method really matter? Psychological Science, 24(10), 1918–1927. 10.1177/0956797613480187 23955356

[jcv212202-bib-0032] Hewitt, O. M. , Tomlin, A. , & Waite, P. (2021). The experience of panic attacks in adolescents: An interpretative phenomenological analysis study. Emotional and Behavioural Difficulties, 26(3), 240–253. 10.1080/13632752.2021.1948742

[jcv212202-bib-0033] Holmes, E. , Brown, R. , Mansell, W. , Fearon, R. , Hunter, E. , Frasquilho, F. , & Oakley, D. (2005). Are there two qualitatively distinct forms of dissociation? A review and some clinical implications. Clinical Psychology Review, 25(1), 1–23. 10.1016/j.cpr.2004.08.006 15596078

[jcv212202-bib-0034] Hunter, E. C. M. , Phillips, M. L. , Chalder, T. , Sierra, M. , & David, A. S. (2003). Depersonalisation disorder: A cognitive–behavioural conceptualisation. Behaviour Research and Therapy, 41(12), 1451–1467. 10.1016/S0005-7967(03)00066-4 14583413

[jcv212202-bib-0035] IBM Corp . (2021). IBM SPSS statistics for windows.

[jcv212202-bib-0036] Kekkonen, V. , Kraav, S.‐L. , Hintikka, J. , Kivimäki, P. , Kaarre, O. , & Tolmunen, T. (2021). Stability of alexithymia is low from adolescence to young adulthood, and the consistency of alexithymia is associated with symptoms of depression and dissociation. Journal of Psychosomatic Research, 150, 110629. 10.1016/j.jpsychores.2021.110629 34598049

[jcv212202-bib-0037] Lantrip, C. , Isquith, P. K. , Koven, N. S. , Welsh, K. , & Roth, R. M. (2016). Executive function and emotion regulation strategy use in adolescents. Applied Neuropsychology: Child, 5(1), 50–55. 10.1080/21622965.2014.960567 25650638

[jcv212202-bib-0038] Loas, G. , Braun, S. , Delhaye, M. , & Linkowski, P. (2017). The measurement of alexithymia in children and adolescents: Psychometric properties of the Alexithymia Questionnaire for Children and the twenty‐item Toronto Alexithymia Scale in different non‐clinical and clinical samples of children and adolescents. PLoS One, 12(5), e0177982. 10.1371/journal.pone.0177982 28542508 PMC5444663

[jcv212202-bib-0039] Lofthouse, M. K. , Waite, P. , & Černis, E. (2023). Developing an understanding of the relationship between anxiety and dissociation in adolescence. Psychiatry Research, 324, 115219. 10.1016/j.psychres.2023.115219 37119790

[jcv212202-bib-0040] Loh, W. W. , & Ren, D. (2023). Adjusting for baseline measurements of the mediators and outcome as a first step toward eliminating confounding biases in mediation analysis. Perspectives on Psychological Science, 18(5), 1254–1266. 10.1177/17456916221134573 36749872

[jcv212202-bib-0041] Majohr, K.‐L. , Leenen, K. , Grabe, H. J. , Jenewein, J. , Nuñez, D. G. , & Rufer, M. (2011). Alexithymia and its relationship to dissociation in patients with panic disorder. The Journal of Nervous and Mental Disease, 199(10), 773–777. 10.1097/NMD.0b013e31822fcbfb 21964271

[jcv212202-bib-0042] Marshall, R. D. , Schneier, F. R. , Lin, S. H. , Simpson, H. B. , Vermes, D. , & Liebowitz, M. (2000). Childhood trauma and dissociative symptoms in panic disorder. American Journal of Psychiatry, 157(3), 451–453. 10.1176/appi.ajp.157.3.451 10698824

[jcv212202-bib-0043] NHS Digital . (2020). Mental health of children and young people in England, 2020: Wave 1 follow up to the 2017 survey. Retrieved April 28, 2022, from https://digital.nhs.uk/data‐andinformation/publications/statistical/mental‐health‐ofchildren‐and‐young‐people‐in‐england/2020‐wave‐1‐follow‐up;https://files.digital.nhs.uk/AF/AECD6B/mhcyp_2020_rep_v2.pdf

[jcv212202-bib-0044] Office for National Statistics (2010). SOC2010 volume 1: Structure and descriptions of unit groups. https://www.ons.gov.uk/methodology/classificationsandstandards/standardoccupationalclassificationsoc/soc2010/soc2010volume1structureanddescriptionsofunitgroups

[jcv212202-bib-0045] Pincus, D. B. , Ehrenreich, J. T. , & Mattis, S. G. (2008). Mastery of anxiety and panic for adolescents: Therapist guide: Riding the wave. Oxford University Press. 10.1093/med:psych/9780195335804.001.0001

[jcv212202-bib-0046] Powers, A. , & Casey, B. J. (2015). The adolescent brain and the emergence and peak of psychopathology. Journal of Infant, Child, and Adolescent Psychotherapy, 14(1), 3–15. 10.1080/15289168.2015.1004889

[jcv212202-bib-0047] Preacher, K. J. , & Hayes, A. F. (2004). SPSS and SAS procedures for estimating indirect effects in simple mediation models. Behavior Research Methods, Instruments, & Computers, 36(4), 717–731. 10.3758/BF03206553 15641418

[jcv212202-bib-0048] Rieffe, C. , Oosterveld, P. , & Terwogt, M. M. (2006). An alexithymia questionnaire for children: Factorial and concurrent validation results. Personality and Individual Differences, 40(1), 123–133. 10.1016/j.paid.2005.05.013

[jcv212202-bib-0049] Šago, D. , Babić, G. , Bajić, Ž. , & Filipčić, I. (2020). Panic disorder as unthinkable emotions: Alexithymia in panic disorder, a Croatian cross‐sectional study. Frontiers in Psychiatry, 11, 466. 10.3389/fpsyt.2020.00466 32581863 PMC7282461

[jcv212202-bib-0050] Seguí, J. , Márquez, M. , García, L. , Canet, J. , Salvador‐Carulla, L. , & Ortiz, M. (2000). Depersonalization in panic disorder: A clinical study. Comprehensive Psychiatry, 41(3), 172–178. 10.1016/s0010-440x(00)90044-0 10834625

[jcv212202-bib-0051] Shear, M. K. , Brown, T. A. , Barlow, D. H. , Money, R. , Sholomskas, D. E. , Woods, S. W. , Gorman, J. M. , & Papp, L. A. (1997). Multicenter collaborative panic disorder severity scale. American Journal of Psychiatry, 154(11), 1571–1575. 10.1176/ajp.154.11.1571 9356566

[jcv212202-bib-0052] Strauss, A. Y. , Kivity, Y. , & Huppert, J. D. (2019). Emotion regulation strategies in cognitive behavioral therapy for panic disorder. Behavior Therapy, 50(3), 659–671. 10.1016/j.beth.2018.10.005 31030881

[jcv212202-bib-0053] Tibubos, A. N. , Grammes, J. , Beutel, M. E. , Michal, M. , Schmutzer, G. , & Brähler, E. (2018). Emotion regulation strategies moderate the relationship of fatigue with depersonalization and derealization symptoms. Journal of Affective Disorders, 227, 571–579. 10.1016/j.jad.2017.11.079 29172049

[jcv212202-bib-0054] Vizard, T. , Pearce, N. , & Davis, J. (2018). Mental health of children and young people in England. Health and Social Care Information Centre.

