# Supplementary material for: The relationship between dissociation and panic symptoms in adolescence and the exploration of potential mediators
Source: JCPP Adv. 2023 Oct 11;4(1):e12202. doi: 10.1002/jcv2.12202 (PMC10933600; doi:10.1002/jcv2.12202)
Supplement: Supplementary file 1 — Supporting Information S1 [file JCV2-4-e12202-s001.docx]

The Relationship Between Dissociation and Panic Symptoms in Adolescence and the Exploration of Potential Mediators

Lottie Shipp, Alisa Musatova, Emma Černis and Polly Waite

**Supporting Information**

**Figure S1.**

*Hypothesised Associations*


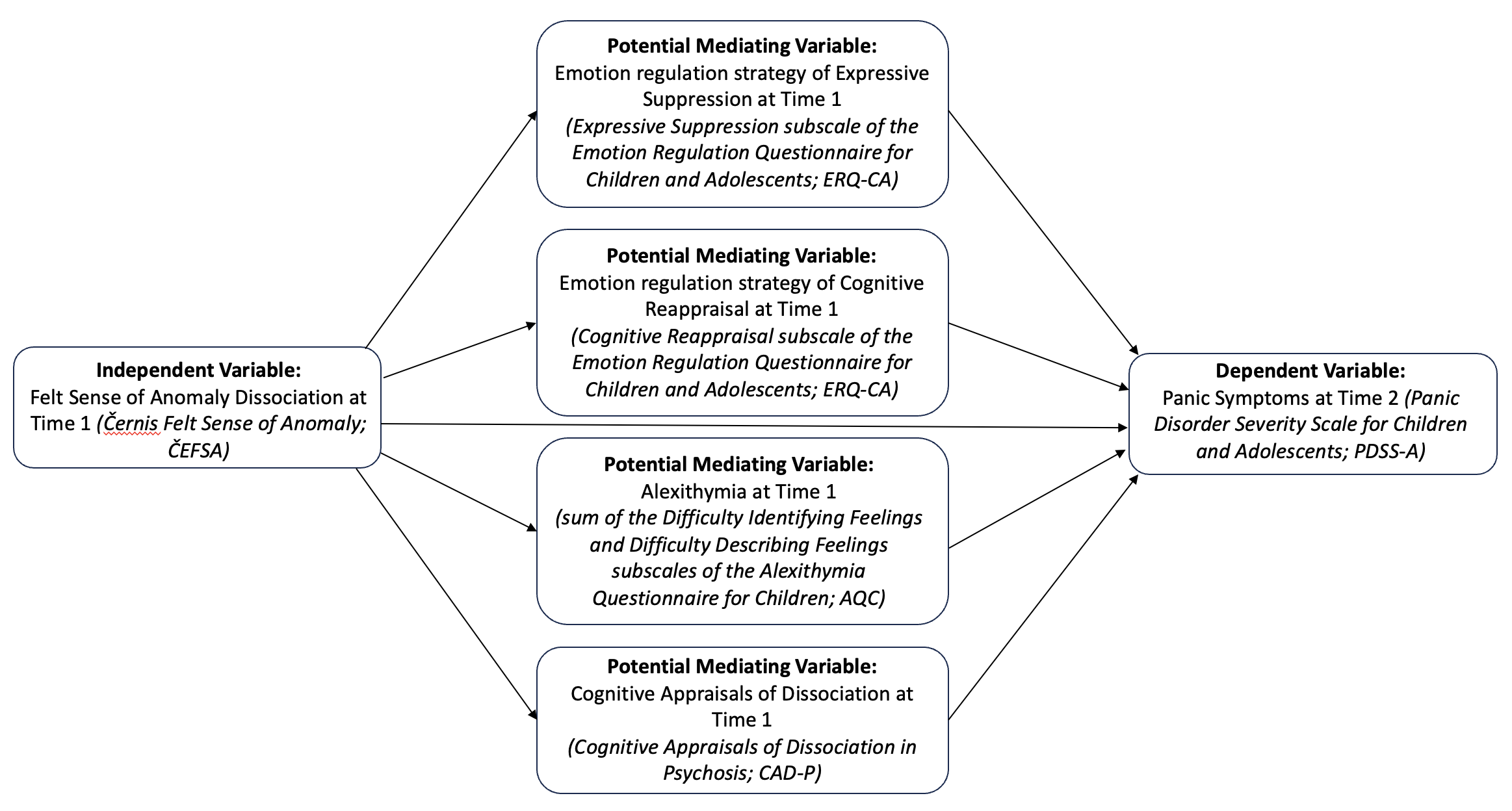


**Figure S2**.

*Participant recruitment and flow through the study*


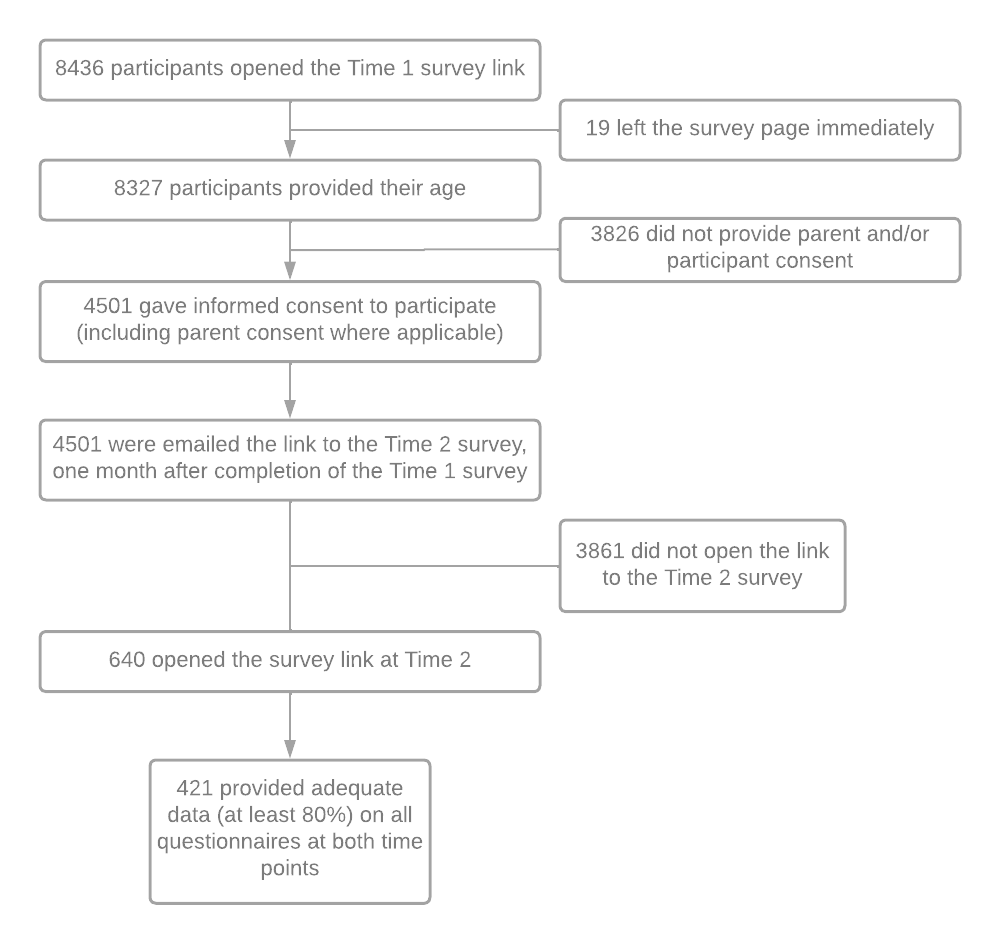


**Figure S3.**

*Study Procedure*


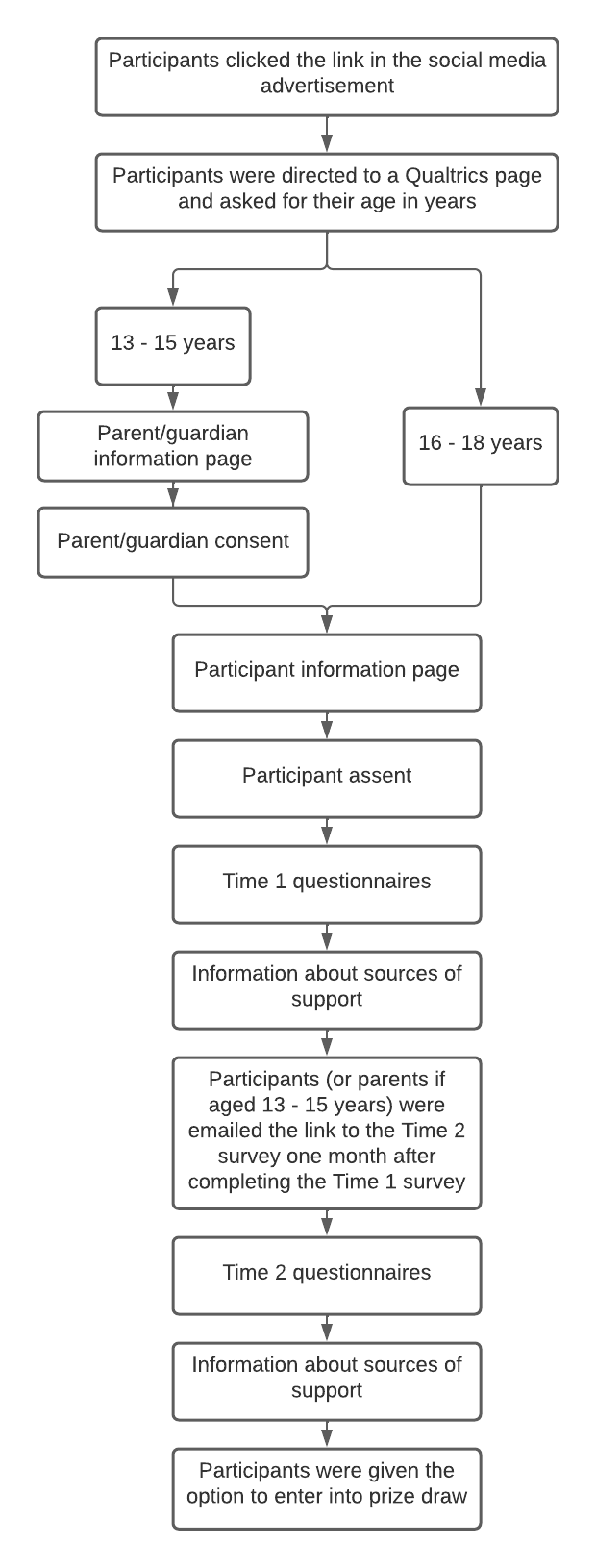


**Table S1**

*FSA-Dissociation Regression and Mediation – Exploratory Analyses Controlling for Panic Symptoms at Time 1*

| ***Regression Analysis*** |  |  |  |  | 95% Confidence Interval | |
| --- | --- | --- | --- | --- | --- | --- |
|  | 𝛽 | Standard Error | *t* | *p* | Lower | Upper |
| FSA-Dissociation on Expressive Suppression | 0.27 | 0.006 | 4.47 | <0.001 | 0.015 | 0.039 |
| FSA-Dissociation on Cognitive Reappraisal | -0.01 | 0.008 | -1.74 | 0.083 | -0.028 | 0.002 |
| FSA-Dissociation on Alexithymia | 0.08 | 0.007 | 11.12 | <0.001 | 0.065 | 0.093 |
| FSA-Dissociation on Cognitive Appraisals of Dissociation | 0.26 | 0.013 | 20.81 | <0.001 | 0.239 | 0.289 |
| Expressive Suppression on Panic Symptoms | 0.10 | 0.059 | 1.61 | 0.109 | -0.021 | 0.212 |
| Cognitive Reappraisal on Panic Symptoms | -0.05 | 0.045 | -1.17 | 0.244 | -0.140 | 0.036 |
| Alexithymia on Panic Symptoms | -0.02 | 0.052 | -0.43 | 0.666 | -0.124 | 0.079 |
| Cognitive Appraisals of Dissociation on Panic Symptoms | 0.04 | 0.027 | 1.38 | 0.168 | -0.016 | 0.090 |
| FSA-Dissociation on Panic Symptoms | 0.02 | 0.010 | 1.96 | 0.050 | 0.000 | 0.040 |
| ***Mediation Analysis*** |  |  |  |  | 95% Confidence Interval | |
|  | Effect | Standard Error | *t* | *p* | Lower | Upper |
| Total Effect of FSA-Dissociation on Panic Symptoms | 0.03 | 0.007 | 4.53 | <0.001 | 0.018 | 0.045 |
| Direct Effect of FSA-Dissociation on Panic Symptoms | 0.02 | 0.010 | 1.96 | 0.050 | 0.000 | 0.040 |
|  |  |  |  |  | Bootstrapped 95% Confidence Interval | |
| *Indirect Effects of FSA-Dissociation on Panic Symptoms* | Effect | Bootstrapped Standard Error | |  | Lower | Upper |
| Via Expressive Suppression | 0.0026 | 0.0017 | |  | -0.0004 | 0.0063 |
| Via Cognitive Reappraisal | 0.0007 | 0.0008 | |  | -0.0005 | 0.0025 |
| Via Alexithymia | -0.0018 | 0.0035 | |  | -0.0087 | 0.0049 |
| Via Cognitive Appraisals of Dissociation | 0.0098 | 0.0082 | |  | -0.0063 | 0.0259 |
